# Monocyte eukaryotic initiation factor 2 signaling differentiates 17-hydroxy-docosahexaenoic acid levels and pain

**DOI:** 10.1016/j.isci.2025.111862

**Published:** 2025-01-21

**Authors:** Peter R.W. Gowler, Asta Arendt-Tranholm, James Turnbull, Rakesh R. Jha, David Onion, Tony Kelly, Afroditi Kouraki, Paul Millns, Sameer Gohir, Susan Franks, David A. Barrett, Ana M. Valdes, Victoria Chapman

**Affiliations:** 1 Pain Centre Versus Arthritis and NIHR Nottingham Biomedical Research Centre, School of Life Sciences, Faculty of Medicine and Health Sciences, University of Nottingham, Nottingham, UK; 2 Centre for Analytical Bioscience, Advanced Materials and Healthcare Technologies Division and NIHR Nottingham Biomedical Research Centre, School of Pharmacy, University of Nottingham, Nottingham, UK; 3 Pain Centre Versus Arthritis and NIHR Nottingham Biomedical Research Centre, School of Medicine, University of Nottingham, Nottingham, UK; 4 School of Mathematical Sciences, University of Nottingham, Nottingham, UK; 5 Flow Cytometry Facility, School of Life Sciences, University of Nottingham, Nottingham, UK

**Keywords:** Biological sciences, Molecular neuroscience, Neuroscience

## Abstract

Our goal was to probe the potential transcriptomic basis for the relationship between plasma levels of the specialized pro-resolving precursor, 17-hydroxy-docosahexaenoic acid (17-HDHA) and chronic pain. Participants with osteoarthritis (average age of 62.3, 60% were female, *n* = 30) were stratified by levels of 17-HDHA and self-reported pain scores. RNAs from CD14++/CD16-/CD66b-/HLA-DR+ (classical) monocytes were sequenced and differentially expressed mRNAs were identified with DESeq2. QIAGEN ingenuity pathway analysis identified the top ranked canonical biological pathway to be eukaryotic initiation factor 2 (EIF2) signaling (lower activation level in the low 17-HDHA-high pain group compared to the high 17-HDHA-low pain group (*Z* score −3)), followed by EIF4 and P70S6K signaling pathways and mTOR signaling. Our approach provides insight into the biological pathways contributing to the association between 17-HDHA and chronic osteoarthritis (OA) pain, identifying EIF2 signaling, with known roles in osteoclast differentiation, OA pathology, and pain, as a potential downstream target.

## Highlights

• Expression levels of PLA2G6 and EPHX2 were associated with levels of 17-HDHA
• eIF2 signaling was the top ranked pathway in classical monocytes between key groups
• The next highly ranked pathways were eIF4, P70S6K, and mTOR signaling

## Graphical abstract

**Figure:**
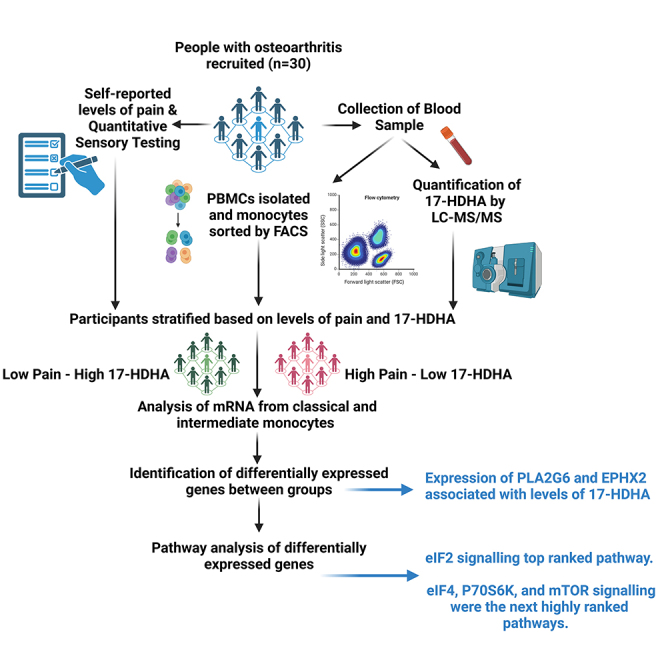

## Introduction

Chronic pain is a global burden, and osteoarthritis (OA) is the fastest growing contributor.^1^ OA involves changes in multiple synovial joint tissues^2^ and variable pain severity.^3^ Joint inflammation is an important pain driver,^4^ but non-steroidal anti-inflammatory drugs have limited therapeutic utility and major long term safety issues.^5^ Dynamic changes to the OA joint include the recruitment of circulating monocytes to the synovium and their differentiation into tissue macrophages,^6^ which have increased plasticity compared to resident macrophages.^7^ Synovial accumulation of pro-inflammatory macrophages is a feature of OA^8^^,^^9^ and the release of pro-inflammatory cytokines and lipids by subsets of recruited and tissue resident macrophages drives OA pathology and the sensitization of sensory nerves and pain.^8^^,^^10^ Peripheral noccieptor sensitization mechanisms include signaling pathways upstream of translation, such as the phosphorylation of PI3K, AkT, MAPKs, mTOR, eIF4E, and s6, which have been associated with inflammatory pain states and the transition to chronic pain.^11^

The oxylipins are derived from omega-3 and -6 polyunsaturated fatty acids (PUFAs) via the cyclooxygenase-2, cytochrome P450, and lipoxygenase enzymatic pathways by multiple cell types.^12^ The resolvins, maresins, lipoxins, and protectins are a class of oxylipins termed “specialised pro-resolving mediators” (SPMs).^13^ SPMs have been suggested to curtail inflammatory responses via multiple mechanisms, including inhibition of nuclear factor kappa B (NF-kB) activation, attenuation of chemokine and cytokine production, and regulation of leukocyte trafficking and clearance.^14^ We reported that higher levels of 17-docosahexaenoic acid (17-HDHA), a precursor to the D-series resolvins, are associated with lower heat pain thresholds in healthy volunteers, and with lower pain scores in OA patients.^15^ A finding also present in a murine model of post-traumatic OA, where we also reported increased numbers of CD68 and CD206 positive macrophages in the murine OA knee joint.^16^ Importantly given the controversy surrounding the SPMs,^17^ exogenous administration with 17-HDHA reduced pain behavior in an experimental model of OA^18^ and exogenous application of the D- and E-series resolvins reduced experimental inflammatory and neuropathic pain responses.^19^^,^^20^^,^^21^ Multiple lines of evidence support 17-HDHA driving an anti-inflammatory macrophage phenotype.^22^^,^^23^^,^^24^ We hypothesized that circulating levels of 17-HDHA and pain may be associated with transcriptomic differences in populations of monocytes.

The aim of this study was to use next-generation sequencing of classical and intermediate monocytes collected from people with varying levels of OA pain and plasma 17-HDHA to undertake an exploratory analysis of the potential contribution of the monocyte transcriptome to this relationship. Using an *priori* analysis of genes involved in the 17-HDHA pathway and inflammatory signaling and an unbiased QIAGEN ingenuity pathway analysis of the differential gene expression, we identified gene expression patterns with known pain and stress response-related biological pathways in the classical monocytes which are implicated in the variance in levels of 17-HDHA and pain in people with OA.

## Results

**Figure 1:**
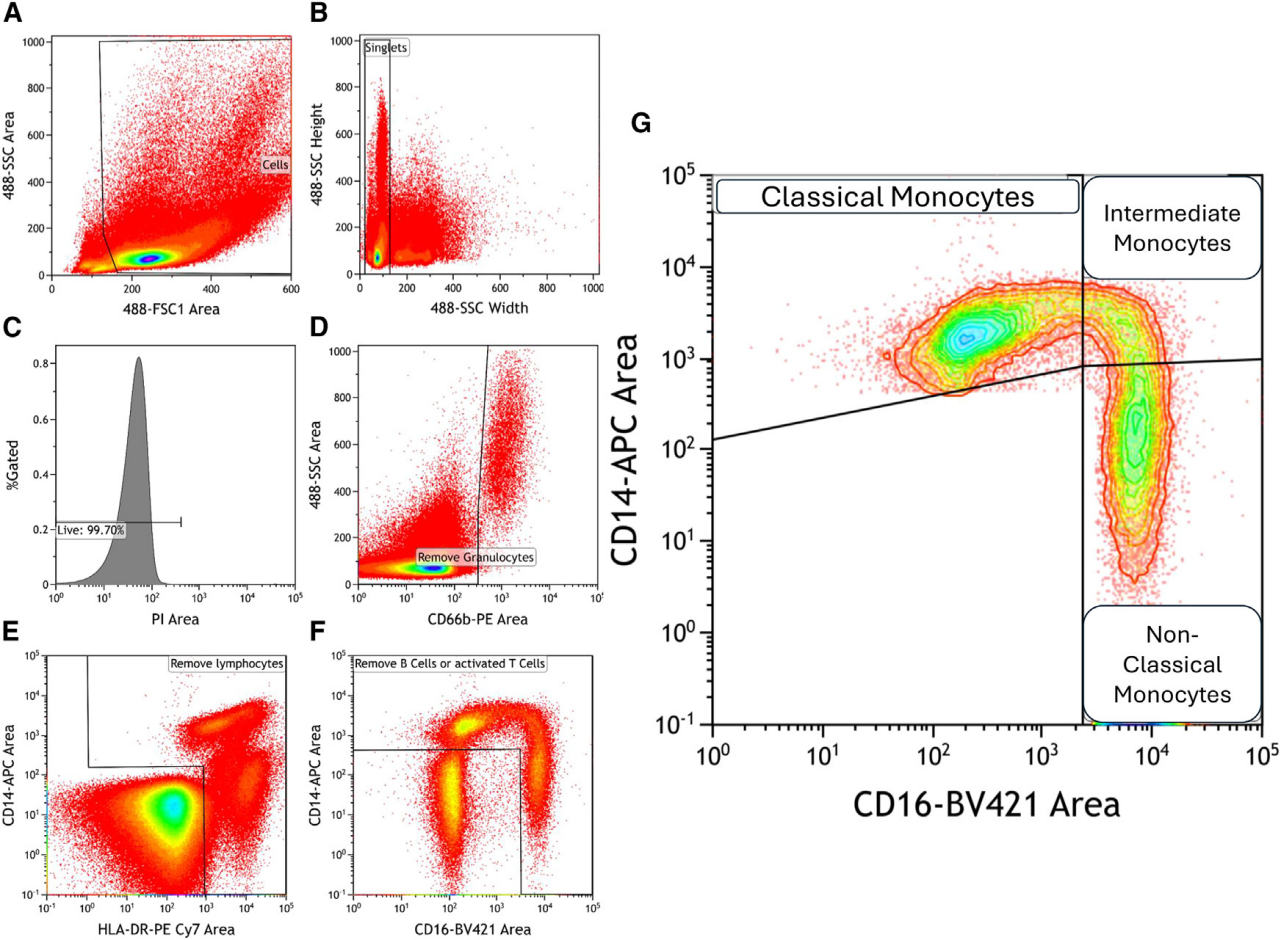
Flow cytometry gating strategy for sorting monocytes Analytical gating of flow cytometry data: (A) PBMCs were gated on a forward scatter (FSC) versus forward scatter (FSC) plot; (B) gating strategy to identify single cells; (C) gated on PI negative cells to determine viable cells; (D) gating strategy to remove granulocytes; (E) gating strategy to remove lymphocytes; (F) gating strategy to remove B cells and activated T cells; and (G) gating of monocyte subsets was performed for classical (CD14+/CD16-), intermediate (C14+/CD16+), and non-classical (CD14-/CD16+) populations.

Self-reported pain, plasma levels of 17-HDHA and mRNA expression in two populations of monocytes were analyzed from 30 people with OA recruited from the iBEAT OA cohort (Figure 1).^25^ Participants in this study had an average age of 62.3, body mass index (BMI) of 30.1, Kellgran-Lawrence (KL) grade of 2, an numerical rating scale (NRS) score of 5, and 60% were female (Table 1). Peripheral blood mononuclear cells (PBMCs) were isolated from whole blood using fluorescence-activated cell sorting (FACS). Monocytes were separated into classical CD14++/CD16-/CD66b-/HLA-DR+ (classical) and CD14++/CD16+/CD66b-/HLA-DR+ 9 intermediate) populations (Figure 1). There were no correlations between the numbers of the two monocyte populations with self-reported pain or 17-HDHA plasma concentration (data not shown).

**Table 1:** Characteristics of the study participants and sub-groups

	All participants	Low 17-HDHA-low pain (LHLP)	High 17-HDHA-low pain (HHLP)	Low 17-HDHA-high pain (LHHP)	High 17-HDHA-high pain (HLHP)
Group size (n)	30	9	8	8	5
Sex (n, % female)	18 (60.0%)	5 (56%)	5 (62.5%)	5 (62.5%)	3 (60.0%)
Age (mean, SD)	62.3 (7.1)	61.2 (2.2)	66.2 (3.0)	61 (2.6)	62 (2.3)
BMI (mean, SD)	30.1 (6.0)	33.2 (2.3)	28.1 (2.5)	27.9 (1.3)	31.1 (1.7)
KL grade (median, IQR)	2 (1–2.8)	2 (1.0–2.5)	2 (1.0–3.0)	1.5 (1.0–3.0)	2 (1.5–2.0)
NRS score (median, IQR)	5 (4–7)	4 (2.5–5.0)	4.3 (2.5–5.0)	7 (6.6–7.8)	7 (6.0–7.5)
Plasma 17-HDHA concentration (nM [mean, SD])	0.43 (0.2)	0.23 (0.03)	0.6 (0.04)	0.33 (0.04)	0.68 (0.05)
Classical monocyte count (median, IQR)	9805 (3378–15598)	13347 (9039–15846)	10887 (4252–18948)	19452 (1297–19452)	5277 (2720–104472)
Intermediate monocyte count (median, IQR)	943 (365–1571)	1083 (968–1681)	571 (383–3959)	377 (162–1179)	524 (218–10954)

BMI, body mass index; KL, Kellgran-Lawrence; NRS, numerical rating scale; 17-HDHA (17-hydroxydocosahexaenoic acid).

### A prior gene analysis in two populations of monocytes

We first focused on an *a priori* set of genes, selected on the basis of established involvement in the synthesis and catabolism of 17-HDHA, the receptors for the resolvins, and OA associated inflammatory molecules. Associations of these genes with plasma levels of 17-HDHA and pain scores were analyzed for the two monocyte populations. For the classical monocytes, the rlog transformed hit counts for PLA2G6, which encodes a phospholipase A2, were correlated with plasma levels of 17-HDHA (Table 2). For the intermediate monocytes, the rlog transformed hit counts for EPHX2, which encodes soluble epoxide hydrolase, were correlated with plasma levels of 17-HDHA (Table 3). None of the *a priori* genes studied in the two monocyte populations were significantly associated with OA pain scores (Tables 2 and 3).

**Table 2:** Correlations between selected genes with NRS and 17-HDHA levels in classical monocytes

	Classical monocytes	Correlation with pain		Correlation with 17-HDHA	
	Gene Name	r	*p* value	r	*p* value
Related to synthesis of resolvins	ALOX12	−0.08869	0.6412	−0.2912	0.1184
	ALOX15B	−0.2845	0.1275	0.2516	0.1798
	ALOX5	−0.05223	0.784	−0.004672	0.9805
	ALOX5AP	−0.05875	0.7578	−0.3264	0.0783
	CYP4F22	−0.07091	0.7096	0.0623	0.7436
	CYP4F3	−0.1189	0.5316	−0.09812	0.606
	CYP4V2	0.2544	0.1749	0.2743	0.1424
	LTA4H	−0.124	0.5137	−0.176	0.3522
	PTGS2	−0.01531	0.936	−0.06408	0.7366
Related to degradation of resolvins	HPGD	0.09973	0.6001	−0.2459	0.1903
Genes for resolvin receptors	CMKLR1	−0.1344	0.4789	0.1738	0.3584
	FPR2	−0.08082	0.6712	−0.03382	0.8592
	LTB4R	−0.2085	0.269	0.02225	0.9071
	LTB4R2	0.2123	0.2601	0.08455	0.6569
Other genes of interest	TBXAS1	−0.1182	0.5339	−0.05473	0.7739
	EPHX1	−0.07451	0.6956	−0.03093	0.8711
	EPHX2	0.2256	0.2307	−0.1618	0.3931
	PLA2G12A	0.01801	0.9247	−0.06252	0.7428
	PLA2G15	0.06618	0.7282	−0.02603	0.8914
	PLA2G4A	−0.06551	0.7309	0.08677	0.6484
	PLA2G4C	0.07181	0.7061	−0.03738	0.8445
	PLA2G6	0.2145	0.2549	0.3702	0.044

A Priori analysis of 22 genes of interest involved in the synthesis, metabolism, and signaling of 17-HDHA in the classical monocyte population (*n* = 30). The relationship between the rlog transformed hit counts of the selected genes and the self-reported pain (NRS) or 17-HDHA concentration were analyzed by Spearman’s Rho with correction for multiple comparison using false discovery rate.

**Table 3:** Correlations between selected genes with NRS and 17-HDHA levels in intermediate monocytes

	Intermediate monocytes	Correlation with pain		Correlation with 17-HDHA	
	Gene name	r	*p* value	r	*p* value
Related to synthesis of resolvins	ALOX12	0.08217	0.666	−0.2961	0.1121
	ALOX15B	0.002476	0.9896	0.1095	0.5647
	ALOX5	0.02544	0.8939	−0.02336	0.9025
	ALOX5AP	0.0959	0.6142	−0.3469	0.0604
	CYP4F22	0.2271	0.2274	0.07209	0.705
	CYP4F3	0.02431	0.8985	−0.1778	0.3473
	CYP4V2	0.06326	0.7398	0.1549	0.4139
	LTA4H	−0.2632	0.16	0.02381	0.9006
	PTGS2	0	0.9999	0.1644	0.3853
Related to degradation of resolvins	HPGD	0.1342	0.4797	−0.1406	0.4586
Genes for resolvin receptors	CMKLR1	−0.1468	0.439	0.2114	0.2622
	FPR2	−0.06461	0.7345	0.2203	0.2421
	LTB4R	−0.06191	0.7452	−0.02447	0.8979
	LTB4R2	0.1997	0.2901	−0.1604	0.3971
Other genes of interest	TBXAS1	−0.3305	0.0745	0.1473	0.4373
	EPHX1	−0.04502	0.8132	−0.3344	0.0709
	EPHX2	0.06033	0.7515	−0.3705	0.0439
	PLA2G12A	0.1916	0.3105	−0.1673	0.3768
	PLA2G15	0.2611	0.1634	−0.1662	0.3801
	PLA2G4A	−0.118	0.5347	0.02981	0.8757
	PLA2G4C	0.05043	0.7913	−0.1279	0.5005
	PLA2G6	0.07519	0.6929	−0.2717	0.1464

A Priori analysis of 22 genes of interest involved in the synthesis, metabolism, and signaling of 17-HDHA in the intermediate monocyte population (*n* = 30). The relationship between the rlog transformed hit counts of the selected genes and the self-reported pain (NRS) or 17-HDHA concentration were analyzed by Spearman’s Rho.

#### Differentially expressed gene expression patterns in two populations of monocytes

The second stage of analysis focused on the gene expression patterns when participants stratified based on their pain scores (low pain <5.9 for NRS, high pain >6 for NRS) and plasma concentrations of 17-HDHA (low 17-HDHA <0.49 nmol, high 17-HDHA >0.5nmol) (Table 1). While these groups had significantly different self-reported pain scores and plasma concentrations of 17-HDHA (Figure S1), other clinical characteristics, including age, sex, BMI, and KL scores were comparable between groups (Table 1). There were no differences in the proportion of the different monocyte populations when expressed as a percentage of total monocytes and percentage of total live cells between the 4 groups (Figure S2).

**Figure 2:**
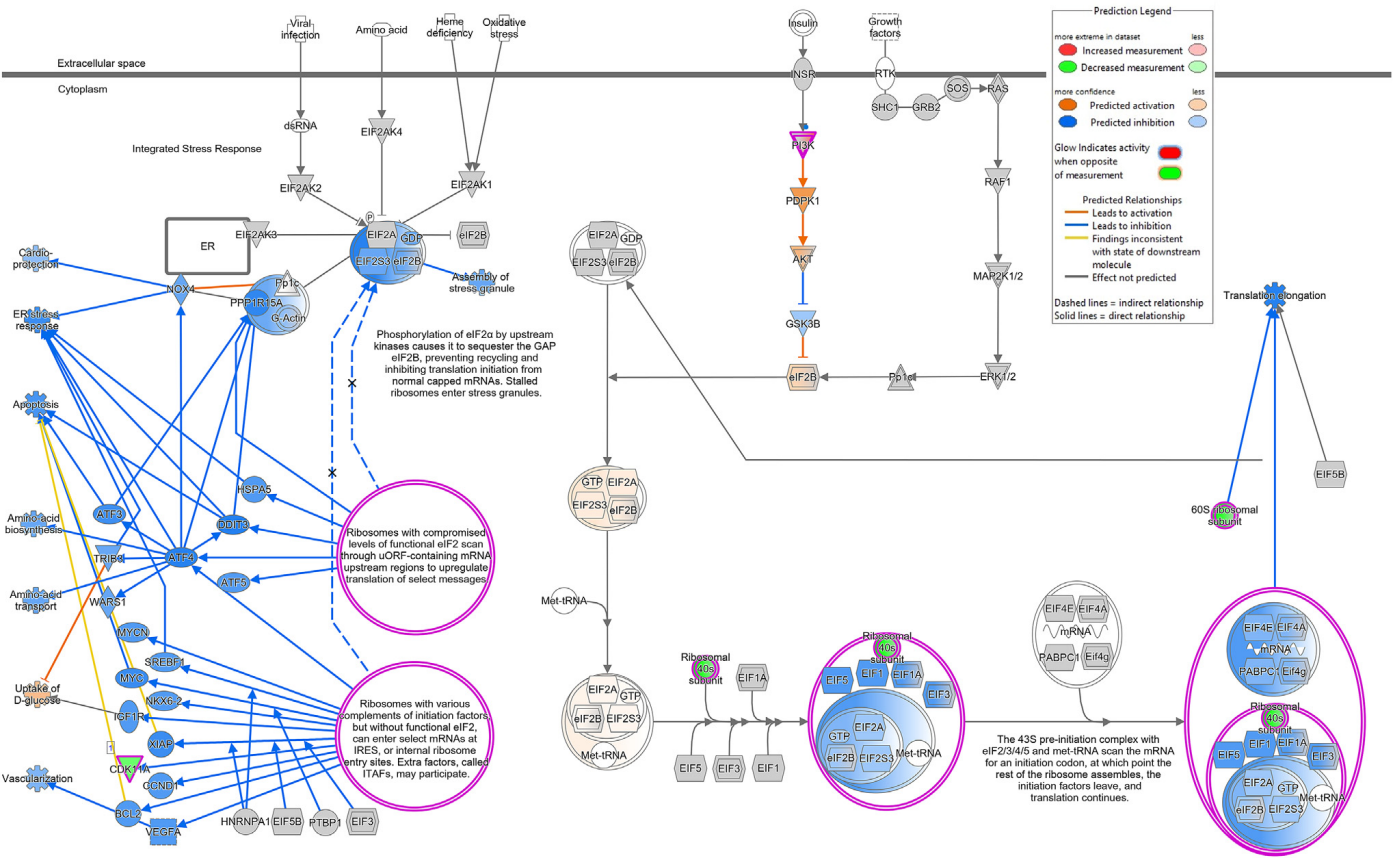
The EIF2 signaling pathway The EIF2 signaling pathway color-coded for the expression pattern of key pathway mediators when comparing the high 17-HDHA and low pain group and the low 17-HDHA and high pain group for the classical monocytes.

The DESeq2 pipeline identified a number of differentially expressed up- and downregulated genes between the different subgroups (Table 4; Table S1) for the classical (Table 5) and intermediate (Table 5) monocytes. For our primary comparison of the groups high 17-HDHA-low pain (HH-LP) versus low 17-HDHA-high pain (LH-HP), 4 differentially expressed genes (DEGs) were identified (adjusted *p* < 0.05) for the classical monocytes (hemoglobin subunit beta [HBB], hemoglobin subunit alpha 1 [HBA1], hemoglobin subunit mu [HBM], and hemoglobin subunit delta [HBD]) (Table 5), and no DEGS were identified for the intermediate monocytes (Table 5). The low number of differentially expressed genes (DEGs) identified are hypothesized to be due to the inherent variability between patient samples coupled with the small patient sample groups. To avoid imposing undue value on individual gene expression patterns, QIAGEN IPA was utilized to investigate any biologically relevant expression patterns. This exploratory analysis was performed using genes with an unadjusted *p* < 0.05 and log2 fold change >1 for the primary comparison of interest (HH-LP versus LH-HP). Ranking of pathways for this comparison for the classical monocytes identified eukaryotic initiation factor 2 (EIF2) signaling as the top ranked pathway (Table 6), which had a lower activation level in the LH-HP group compared to the HH-LP group (*Z* score −3). The genes making up the EIF2 signaling pathway for the HH-LP and LH-HP comparison are described in Table 7 and the direction and expression pattern are shown graphically in Figure 2. Key elements of the EIF2 signaling pathway included the predicted activation of PDPK1 and AKT upstream of eIF2B (Figure 2). Other elements of the EIF2 signaling pathway (including CDK11A and multiple RPL ribosomal proteins), which drive changes to signaling related to translation initiation of select RNAs, were predicted to have a lower activation status (Figure 2). To assess whether the predicted changes to the EIF2 signaling pathway reflected transcriptomic differences specific to the relationship between levels of 17-HDHA and pain, the transcriptomic variation associated with pain irrespective of levels of 17-HDHA was also determined. The gene expression patterns identified in the classical monocytes for high pain alone included two genes (CDK11A and RPS8) with a *p* value < 0.05, that contributed to EIF2 signaling pathway identified in our primary comparison of interest (Table 7). For the transcriptomic variation associated with levels of 17-HDHA alone, one gene (RPS26) with a *p* value < 0.05 overlapped with the genes contributing to the EIF2 signaling pathway identified in our primary comparison of interest (Table 7).

**Table 4:** The number of upregulated and downregulated differentially expressed genes (DEGs) identified with DESeq2 for comparisons between groups for the classical and intermediate monocytes for the 4 pre-defined groups (Log2 (fold-change) adjusted *p* < 0.05)

Group	Comparison	Upregulated	Downregulated	Total DEGs
Classical monocytes	HH-LP vs. LH-HP	0	4	4
	HH-LP vs. HH-HP	1	15	16
	HH-LP vs. LH-LP	0	0	0
	HH-HP vs. LH-HP	22	2	24
	HH-HP vs. LH-LP	10	1	11
	LH-LP vs. LH-HP	3	7	10
Intermediate monocytes	HH-LP vs. LH-HP	0	0	0
	HH-LP vs. HH-HP	3	2	5
	HH-LP vs. LH-LP	5	3	8
	HH-HP vs. LH-HP	26	12	38
	HH-HP vs. LH-LP	2	0	2
	LH-LP vs. LH-HP	12	17	29

HH, high 17-HDHA; LH, low 17-HDHA; HP, high pain; LP, low pain.

**Table 5:** Identification of differentially expressed genes

	HHLPvLHHP	HHLPvHHHP	HHLPvLHLP	HHHPvLHHP	HHHPvLHLP	LHLPvLHHP
Classical monocytes	HBB (−2.19) ↓	C21orf33 (2.37) ↑		PI3∗ (5.27) ↑	TREML1∗ (4.30)	G0S2 (2.97)
	HBA1 (−2.80) ↓	HIST1H2AC∗ (-1.66) ↑		TREML1∗ (5.11) ↑	CAVIN2∗ (3.68)	OSM (2.12)
	HBM∗ (-2.90) ↓	CLU∗ (-2.50) ↓		CTTN (5.11) ↑	ITGB3∗ (3.57)	ENO2 (1.47)
	HBD (−3.21) ↓	TUBB1∗ (-2.84) ↓		SPX (4.99) ↑	CXCL5∗ (3.54)	PEA15 (−1.52) ↓
		HIST1H3H∗ (-2.94) ↓		TREML4∗ (4.90) ↑	TUBB1∗ (3.26)	RN7SL2 (−2.30) ↓
		PDZK1IP1 (−3.08) ↓		ITGB3∗ (4.55) ↑	GNG11∗ (3.20)	RN7SL3 (−2.38) ↓
		ITGA2B∗ (-3.17) ↓		CA4 (4.54) ↑	ENKUR (3.01)	RN7SL5P (−2.47) ↓
		CAVIN2∗ (-3.22) ↓		CXCL5∗ (4.50) ↑	MYL9∗ (2.95)	AL139099.4 (−2.47) ↓
		CXCL5∗ (-3.43) ↓		GP9∗ (4.34) ↑	ITGA2B∗ (2.90)	HBM∗ (-3.92) ↓
		PPBP∗ (-3.60) ↓		BTNL8 (4.25) ↑	HIST1H3H∗ (2.45)	IGLV6-57 (−5.01) ↓
		GP9∗ (-3.64) ↓		LINC00989 (4.06) ↑	AC243829.2 (−2.80) ↓	
		GNG11∗ (-3.92) ↓		PPBP∗ (4.04) ↑		
		TREML1∗ (-4.14) ↓		CAVIN2∗ (3.77) ↑		
		MYL9∗ (-4.24) ↓		GNG11∗ (3.68) ↑		
		PI3∗ (-4.37) ↓		ITGA2B∗ (3.65) ↑		
		TREML4∗ (-5.89) ↓		MFAP3L (3.27) ↑		
				CLU∗ (3.20) ↑		
				ADGRG3 (3.13) ↑		
				SPARC (3.13) ↑		
				MEIS1 (2.51) ↑		
				SELP (2.25) ↑		
				HIST1H2AC∗ (2.11) ↑		
				TTYH3 (−1.31) ↓		
				KHSRP (−2.26) ↓		
Intermediate monocytes		IGLV3-25∗ (5.43) ↑	IGHV3-15∗ (4.52) ↑	SPX∗ (6.62) ↑	SPX∗ (5.88) ↑	AOC2 (3.07) ↑
		IGHV1-24 (4.84) ↑	IGLV3-25∗ (4.32) ↑	LTBP1∗ (5.00) ↑	LTBP1∗ (4.27) ↑	G0S2 (3.02) ↑
		IGHV2-5 (4.43) ↑	IGHV1-3 (4.07) ↑	CXCL5 (4.91) ↑		CXCL8 (2.81) ↑
		PF4∗ (-3.74) ↓	IGHV3-33 (3.70) ↑	GP9 (4.79) ↑		MME (2.60) ↑
		PPBP∗ (-3.81) ↓	IGLV3-9 (2.53) ↑	ITGB3 (4.63) ↑		AC099489.1 (2.36) ↑
			CLEC12A (−1.17) ↓	BEND2 (4.28) ↑		AC012645.1∗ (2.19) ↑
			CLEC1B (−1.80) ↓	SH3BGRL2 (4.13) ↑		ADGRG3∗ (2.04) ↑
			AL512646.1 (−3.40) ↓	PPBP∗ (4.10) ↑		MMP25∗ (2.01) ↑
				PF4∗ (3.90246093) ↑		AC136475.9 (1.95) ↑
				TMEM40 (3.88) ↑		MGAM∗ (1.82) ↑
				KY (3.83) ↑		TNFAIP6 (1.70) ↑
				CAVIN2 (3.77) ↑		AVIL (1.09) ↑
				SPARC (3.48) ↑		CD22∗ (-1.95) ↓
				TUBB1 (3.44) ↑		IGHD∗ (-3.01) ↓
				AC012645.1∗ (3.06) ↑		IGHV3-15 (−3.22) ↓
				ADGRG3∗ (2.95) ↑		IGHV4-34∗ (-3.52) ↓
				KRT23 (2.86) ↑		IGKV2-30 (−3.55) ↓
				MMP25∗ (2.63) ↑		IGLC3 (−3.58) ↓
				MGAM∗ (2.63) ↑		IGLV2-11 (−3.67) ↓
				KCNJ15 (2.61) ↑		IGLV1-47 (−3.68) ↓
				LUCAT1 (2.09) ↑		IGLV1-51∗ (-3.71) ↓
				VNN3 (2.07) ↑		IGKV3-11 (−3.93) ↓
				LRG1 (1.85) ↑		IGKV1D-39∗ (-3.97) ↓
				DGAT2 (1.51) ↑		IGHV1-69D (−4.13) ↓
				MXD1 (1.39) ↑		IGKV1-39 (−4.18) ↓
				CSF3R (1.03) ↑		IGHV3-7 (−4.70) ↓
				PPP1R17 (−1.07) ↓		IGKV3D-20 (−5.53) ↓
				STAP1 (−1.53) ↓		IGHV2-26 (−5.63) ↓
				CD22∗ (-1.75) ↓		IGKV2D-29 (−5.78) ↓
				IGHD∗ (-1.79) ↓		
				IGLV1-51∗ (-2.73) ↓		
				IGKV1-39∗ (-3.34) ↓		
				APOBEC3B (−3.50) ↓		
				IGHV1-18 (−3.66) ↓		
				IGKV1-8 (−4.05) ↓		
				IGHV4-34∗ (-4.21) ↓		
				IGHV4-4 (−4.50) ↓		

DEGs (differentially expressed genes) identified with DESeq2 for each of the 6 comparisons of interest for the classical and intermediate monocyte populations (adjusted *p* value < 0.05). Log2(fold-change) are listed in brackets with cut-offs of more than 1.00 or less than −1.00. Arrows indicate increase (↑) or decrease (↓) in log2(fold-change). DEGs with an asterisk (∗) appear in multiple comparisons.

**Table 6:** Pathway analysis of differentially expressed genes

	Ingenuity Canonical Pathways	-log(*p*-value)	Ratio
Classical monocytes	EIF2 Signaling	19.1	0.147
	Regulation of eIF4 and p70S6K Signaling	9.54	0.112
	mTOR Signaling	8.25	0.0943
	Coronavirus Pathogenesis Pathway	6.36	0.0837
	Communication between Innate and Adaptive Immune Cells	3.57	0.0366
	Basal Cell Carcinoma Signaling	3.34	0.0972
	Hematopoiesis from Pluripotent Stem Cells	3.01	0.0431
	Nitric Oxide Signaling in the Cardiovascular System	2.67	0.0672
	Systemic Lupus Erythematosus Signaling	2.65	0.037
Intermediate Monocytes	Communication between Innate and Adaptive Immune Cells	6.34	0.0312
	Hematopoiesis from Pluripotent Stem Cells	5.68	0.0408
	Systemic Lupus Erythematosus Signaling	5.25	0.0338
	Phospholipase C Signaling	5.23	0.0326
	Autoimmune Thyroid Disease Signaling	4.92	0.0375
	Allograft Rejection Signaling	4.56	0.0352
	Dendritic Cell Maturation	4.01	0.0306
	Regulation of IL-2 Expression in Activated and Anergic T Lymphocytes	3.72	0.0325
	CTLA4 Signaling in Cytotoxic T Lymphocytes	3.55	0.0314

Ingenuity Pathway Analysis (IPA) of DEGs when comparing the high 17-HDHA and low pain group with the low 17-HDHA and high pain group for the classical and intermediate monocytes. For this exploratory analysis DEGS which fulfill the following were inputted into IPA (Log2 fold-change>1 and unadjusted *p* < 0.05).

**Table 7:** List of genes involved in identified signaling pathways

EIF2 Signaling	Regulation of eIF4 and p70S6K Signaling	mTOR Signaling
CDK11A	FAU	FAU
FAU	ITGB5	PIK3C2B
PIK3C2B	PIK3C2B	PIK3R2
PIK3R2	PIK3R2	RPS10
RPL21	RPS10	RPS11
RPL22	RPS11	RPS12
RPL23A	RPS12	RPS13
RPL27	RPS13	RPS14
RPL27A	RPS14	RPS15A
RPL30	RPS15A	RPS16
RPL32	RPS16	RPS17
RPL34	RPS17	RPS18
RPL35A	RPS18	RPS19
RPL36AL	RPS19	RPS20
RPL39	RPS20	RPS23
RPLP1	RPS23	RPS25
RPS10	RPS25	RPS26
RPS11	RPS26	RPS27L
RPS12	RPS27L	RPS8
RPS13	RPS8	VEGFB
RPS14		
RPS15A		
RPS16		
RPS17		
RPS18		
RPS19		
RPS20		
RPS23		
RPS25		
RPS26		
RPS27L		
RPS8		
UBA52		

Gene involved in the regulation of eIF2, elF4 and p70S6K, and mTOR signaling which were identified by ingenuity pathway analysis of differentially expressed genes in classical monocytes between the Low 17-HDHA-High Pain group versus the High 17-HDHA-Low Pain group.

The next two top ranked pathways identified by this exploratory analysis of the comparison of HH-LP versus LH-HP in classical monocytes were EIF4 and P70S6K signaling pathways and mTOR signaling (Table 6), *Z* scores however were not available for these pathways and therefore the direction of effects are unknown. The genes making up these two pathways for the HH-LP and LH-HP comparison in the classical monocytes are described in Table 7.

QIAGEN IPA was also utilized to investigate any biologically relevant expression patterns between the HH-LP versus LH-HP groups for the intermediate monocyte population. In this case, the top ranked pathways were communication between innate and adaptive immune cells and hematopoiesis from pluripotent stem cells and phospholipase signaling (Table 6). A comprehensive comparison of the pathways identified by IPA for the classical and intermediate monocytes for the HH-LP versus LH-HP groups is provided in supplemental information (Figure S3; Table 2). None of the top 8 pathways identified in the classical monocytes were present in the comparison of the same groups for the intermediate monocytes (Table 6). Overlapping pathways between the two monocyte populations for the comparison of interest included communication between innate and adaptive immune cells, hematopoiesis from pluripotent stem cells, CCR5 signaling in macrophages, IL15 signaling and multiple other pathways with known roles in osteoarthritis and rheumatoid arthritis (Table S2).

## Discussion

The present study explored the potential transcriptomic basis for the relationship between plasma levels of 17-HDHA and OA pain. An *a priori* analysis of selected genes identified that the expression levels of two genes (PLA2G6 and EPHX2) were associated with plasma levels of 17-HDHA. None of the *a priori genes* were associated with self-reported pain scores. An unbiased DESeq2 analysis of the transcriptomic datasets for the classical and intermediate monocytes identified DEGs for the key comparison of interest (high 17-HDHA-low pain versus low 17-HDHA-high pain). An exploratory QIAGEN IPA analysis of gene expression patterns identified EIF2 signaling as the top ranked pathway for the classical monocytes for this comparison, with levels of signaling via this pathway predicted to be lower in the low 17-HDHA high pain group. EIF2 signaling has known roles in osteoclast differentiation, which contributes to both OA pathology and pain. The next two ranked pathways for the classical monocytes were EIF4 and P70S6K signaling pathways and mTOR signaling, both of which contribute mechanistically to chronic pain. These top ranked were not identified for the equivalent comparison for the intermediate monocyte population, which was predominantly composed of immune function pathways. The differences in ranked pathways for the two populations of circulating monocytes in this study likely reflect their distinct biological roles following differentiation. Once recruited to the synovium, monocytes are exposed to signals in the joint which stimulate their differentiation into immune cells which can range in phenotype. Classical (CD14^high^) monocytes can differentiate further into pro-inflammatory macrophages and osteoclasts, contributing to synovial tissue inflammation and bone erosion, whereas intermediate (CD14^high^CD16^high^) monocytes can differentiate into pro-inflammatory macrophages contributing to tissue inflammation.^26^ As circulating and synovial monocytes are likely to be transcriptionally distinct, this study highlights the importance of the systemic differences in the monocyte transcriptome between people with varying levels of pain and pro-resolution molecules, which may influence subsequent synovial monocyte functionality, monocyte-derived immune cell phenotype, and their contribution to OA pain and pathology.

### Associations between 17-HDHA and a priori selected genes in OA monocytes

OA is a complex disease involving multiple compartments of joint pathology, including cartilage loss, synovial inflammation, infiltration of mononuclear blood cells into the synovium, subchondral bone remodeling, and sprouting of sensory nerves.^27^ Clinically OA pain is associated with joint inflammation which is characterized by increased numbers of synovial macrophages with a pro-inflammatory phenotype which are both infiltrating and tissue resident.^8^^,^^9^^,^^28^ An *a priori* analysis for the classical monocytes identified that plasma levels of 17-HDHA were associated with hit counts for *PLA2G6*, which encodes phospholipase A2 a key enzyme regulating the release of membrane bound PUFAs into circulation in response to inflammatory signals.^29^ A limitation of the study is the lack of protein samples from these cell preparations which prevented the assessment of protein levels of PLA2G6 in the high vs. low 17-HDHA groups. Plasma levels of 17-HDHA were also associated with *EPHX2* (encodes soluble epoxide hydrolase) expression in intermediate monocytes. Soluble epoxide hydrolase (sEH) converts CYP450 derived anti-inflammatory epoxides (e.g., EETs) into their corresponding diols (e.g., DHETs). Inhibition of sEH has anti-inflammatory and anti-nociceptive effects^30^ and regulates monocyte/macrophage plasticity.^31^ Pharmacological inhibition of sEH stimulates the production of some SPMs, including 17-HDHA, in an experimental inflammatory model,^32^ supporting a link between activity of the sEH pathway and levels of 17-HDHA. Importantly, there are now multiple lines of evidence for associations between the sEH pathway and OA pain,^33^ radiographic knee OA,^34^ and persisting knee pain following traumatic injury in a young cohort.^35^

### Pathway analysis of differentially expressed genes in OA monocytes

QIAGEN IPA analysis identified eIF2 signaling as the top ranked pathway in the classical monocyte population, this pathway had a lower activation level in the low 17-HDHA-high pain group compared to the high 17-HDHA-low pain group. Phosphorylation of eIF2a is downstream of four kinases which protect against cellular stresses including endoplasmic reticulum (ER) stress, oxidation stress, inflammation, and plays a pivotal role in translation inhibition to maintain cellular homeostasis in many cell types, including macrophages and osteoclasts, which are relevant to OA pathology and pain.^36^ Dysregulation of eIF2 signaling has been implicated in multiple complex disease pathologies.^36^ Macrophage stress responses include mRNA translation via phosphorylation of translation initiation factors including eIF2, eIF4, and 4E-BPs.^37^ Subchondral bone remodeling and increased osteoclastogenesis is recognized as an important component of OA pathology and the associated pain.^38^^,^^39^^,^^40^^,^^41^ Bone is heavily innervated, and bone resorption generates a localized acidic environment which can activate sensory nerves.^42^ Blocking osteoclast activity can prevent and reverse OA-like pain behavior in rodent models of OA.^43^^,^^44^ Elevated phosphorylation of eIF2a, as predicted for the high 17-HDHA-low pain group, reduces osteoclast differentiation from bone marrow-derived macrophages,^45^ which would be expected to slow bone remodeling and in the context of OA pain would indirectly reduce pain signaling. Elevated phosphorylation of eIF2a also drives osteoblastogenesis,^46^ which positively influences bone remodeling. Functional roles of eIF2a signaling in OA are supported by the demonstration that inhibition of dephosphorylation of eIF2a reduces cartilage pathology^46^ and synovitis score in a model of OA, however pain responses were not assessed. Overall the predicted lower activation of eIF2a signaling pathways in the low 17-HDHA-high pain group aligns with the known functional role(s) of this pathway in regulating macrophage responses and osteoclast differentiation and subsequently pain.

EIF2 signaling has also been shown to play a role in thermal and inflammatory nociception in sensory nerves.^47^ In this study of acute nociception, decreased eIF2a phosphorylation in transgenic mice reduced behavioral responses to thermal and inflammatory pain stimuli, which was mediated via the pro-nociceptive TRPV1 channel.^47^ In experimental models, blockade of TRPV1 attenuates OA-like pain behavior,^48^^,^^49^ consolidating the link between TRPV1 and OA pain. Although it is established that TRPV1 expression is upregulated in the synovium from people with painful OA^48^ and polymorphisms in TRPV1 are associated with OA pain,^50^ there is an opposing directionality in the role of EIF2 signaling in acute nociception versus in our clinical OA cohort. These differences likely reflect the dynamic interplay between the direct and indirect roles of eIF2 signaling both in maintaining homeostasis in multiple tissue types and modulating differing pain mechanisms, which are collectively involved in complex diseases such as chronic OA pain. Our findings build upon mounting evidence that EIF2 signaling may offer opportunities for novel therapeutic strategies.

The next two highly ranked pathways identified in the primary comparison for the classical monocytes were eIF4 and p70 ribosomal S6 kinase (p70S6K) signaling, as well as mTOR signaling, both of which have established roles in mRNA translation specifically associated with pain processing, macrophage responses and osteoclast differentiation.^9^^,^^37^^,^^51^^,^^52^^,^^53^ mTOR is a serine/threonine kinase activated by multiple signaling cascades in response to tissue damage.^52^^,^^54^ mTOR phosphorylates p70s6K and eukaryotic initiation factor 4E-binding protein 1 (4E-BP1) to regulate protein synthesis^55^ and is downstream of key neurotrophin signaling pathways, including TrKA and TrKB, which have established roles in regulating peripheral sensory nerve excitability in OA and pain hypersensitivity. Nociceptor-specific deletion of 4E-BP1, an mTORC1 downstream effector that represses translation initiation, was sufficient to cause mechanical hypersensitivity.^56^ mTOR, eIF4, p70S6K, and eIF2 have all been characterized as key mediators of translation and protein synthesis in stress responses.^52^ mTOR inhibition attenuated pain responses in models of inflammatory^57^ chronic pain,^58^^,^^59^ and increased levels of phosphorylated mTOR and p70S6K have been demonstrated in DRG and spinal cord neurones,^60^ and the insular cortex^53^ in models of chronic pain.

The eIF2, eIF4, P70S6K, and mTOR signaling pathways are intricately linked, with mTOR serving as a central regulator of protein synthesis and cellular responses to stress, which are particularly relevant in the context of inflammatory conditions, such as OA and pain.^61^ eIF2 initiates translation and its phosphorylation is a key regulatory mechanism that can inhibit protein synthesis under stress conditions, including OA.^62^ The eIF4 complex, which includes eIF4E, eIF4G, and eIF4A, is essential for the recruitment of mRNA to the ribosome,^63^ and mTORC1 promotes the phosphorylation of 4E-BP1, which releases eIF4E and enhances translation initiation.^64^ During conditions of increased inflammatory cytokine production and other stress responses, such as in OA, the regulation of eIF2 and eIF4 by mTOR may influence the synthesis of pro-inflammatory mediators and pain-related proteins. P70S6K, another downstream target of mTOR, regulates ribosomal biogenesis and protein synthesis. Activation of the mTOR pathway leads to the phosphorylation of P70S6K, which further stimulates the translation of mRNAs which mediate cell growth and proliferation.^55^ In OA, the dysregulation of these pathways may lead to enhanced expression of inflammatory cytokines and other mediators driving pain, contributing to the pathophysiology of the disease.

### Limitations of the study

In order to consider the generalizability of these findings, it is important to consider the study design limitations. A total of *n* = 30 highly clinically phenotyped participants were included in this study and were stratified into four groups based on pain scores and plasma levels of 17-HDHA. This resulted in relatively modest and uneven group sizes, which may have skewed analysis of expressed transcripts, nevertheless the different groups were well matched for size, sex, age, BMI, and KL grade which should reduce the impact of heterogeneity of these characteristics within the study participants. Our transcriptomic analysis of the two populations of monocytes from these participant groups has yet to be replicated and therefore further studies in larger OA cohorts, and in other chronic pain conditions will provide greater insight into the reproducibility and generalizability of these transcriptomic findings. The cut-offs used to stratify participants by 17-HDHA and NRS pain score were based on the median values of participants within this study rather than pre-defined values. As a result for different cohorts of OA participants, these cut-offs may change; however, a similar analysis with larger sample size identified the same median NRS cut-off for a community cohort of people with knee pain and OA,^65^ and reported comparable levels of 17-HDHA.^66^ Although in this study the group sizes were well matched for sex, it is a limitation that our study is not adequately powered to consider sex as confounding variable. A previous study has reported sex differences in monocyte transcriptional profiles, specifically highlighting changes to biological pathways identified from gene expression patterns^67^ and sex specific changes to the transcriptional profile of monocytes driven by EPA and DHA has been reported.^68^

### Conclusions

In conclusion in *a priori* analysis expression levels of two genes (PLA2G6 and EPHX2) were associated with plasma levels of 17-HDHA, whereas none of the *a priori genes* were associated with self-reported OA pain scores. An exploratory QIAGEN IPA analysis of the differentially expressed genes identified EIF2 signaling as the top ranked pathway for the classical monocytes, with this pathway predicted to be lower in the low 17-HDHA high pain group. This finding aligns to the known role of EIF2 signaling in osteoclast differentiation, which contributes to both OA pathology and pain. The next two ranked pathways for the classical monocytes were EIF4 and P70S6K signaling pathways and mTOR signaling, both of which contribute mechanistically to chronic pain.

## Resource availability

### Lead contact

Further information and requests for resources should be directed to the lead contact, Prof. Victoria Chapman (victoria.chapman@nottingham.ac.uk).

### Materials availability

This study did not generate new materials.

### Data and code availability

Data will be released via standard procedures overseen by the University of Nottingham and the Nottingham NIHR BRC and its ethical guidelines. Raw sequencing files are available on the European Nucleotide Archive: E-MTAB-14402. Annotated mRNA expression counts, monocyte cell counts, plasma lipidomic measurements, and associated anonymized participant clinical characteristics (age, sex, BMI, KL, and NRS scores) are available upon request. Please contact the corresponding author to receive the application form.

## Acknowledgments

The authors would like to thank Sonal Henson for her assistance in handling the transcriptomic datasets. This research was funded by 10.13039/501100012041Versus Arthritis (grants: 20777 and 21960) and the 10.13039/501100020624NIHR Nottingham Biomedical Research Centre.

## Author contributions

T.K., A.K., and S.G. collected clinical data and biological samples. P.R.W.G., P.M., and D.O. isolated monocytes from whole blood. J.T., R.R.J., and D.A.B. performed lipidomic analysis. P.R.W.G., A.A.-T., J.T., R.R.J., S.F., A.M.V., and V.C. performed data analysis. V.C. and A.M.V. acquired funding, designed the study, and supervised sample collection and analyses of data. All authors contributed to preparation of the manuscript and approved the final version.

## Declaration of interests

The authors declare no competing interests.

## STAR★Methods

### Key resources table

**Table:** 

REAGENT or RESOURCE	SOURCE	IDENTIFIER
**Antibodies**		

APC anti-human CD14	Biolegend	Cat#301807; RRID: AB_314189
Brilliant Violet 421 anti-human CD16	Biolegend	Cat#302037; RRID: AB_10898112
PE anti-human CD66	Biolegend	Cat#342303; RRID: AB_1626288
PE/Cy7 anti-human HLA	Biolegend	Cat#327017; RRID: AB_2566388
Propidium Iodide	Biolegend	Cat#421301; NA

**Biological samples**		

Human Blood	i-BEAT OA Trial	https://pubmed.ncbi.nlm.nih.gov/31662373/ (NCT03545048).

**Chemicals, peptides, and recombinant proteins**		

17-HDHA Reference Standard	Cayman Chemicals	Cat#33650
Lymphoprep	StemCell Technologies	Cat#07851

**Critical commercial assays**		

SMART-Seq v4 Ultra Low Input RNA Kit	Genewiz	https://www.novogene.com/eu-en/services/research-services/transcriptome-sequencing/mrna-sequencing/

**Deposited data**		

RNASeq sequencing and expression files	European Nucleotide Archive	E-MTAB-14402

**Software and algorithms**		

Kaluza Analysis software	Beckman Coulter	https://www.mybeckman.uk/flow-cytometry/software/kaluza
PRISM (V9.0)	GraphPad	https://www.graphpad.com/features
Ingenuity Pathway Analysis	QIAGEN	https://digitalinsights.qiagen.com/products-overview/discovery-insights-portfolio/analysis-and-visualization/qiagen-ipa/
R (V 4.2.0)	R Project	https://www.r-project.org/

### Experimental model and study participant details

Blood samples for this study were provided by 30 participants (Table 1) from the iBEAT-OA (Trial Registration number: NCT03545048, https://clinicaltrials.gov/study/NCT03545048) study,^25^ investigating the effects of six-week exercise intervention on levels of pain. For the majority of participants samples and pain measurements were collected at baseline (n=27) and the rest (n=3) were collected following the intervention. Ethical approval was obtained from the Research Ethics Committee (ref:18/EM/0154) and the Health Research Authority (protocol no: 18021). Participants were enrolled in this trial if they had a numerical rating scale (NRS) score for knee pain of 3 or higher out of 10, and a Kellgren-Lawrence radiographic grade of 1 or higher. Whole blood (12 mL) was collected from these participants for further analysis by mass spectrometry, flow cytometry, and RNA sequencing.

### Method details

#### Quantification 17-HDHA in plasma

Targeted Liquid Chromatography with Tandem Mass-Spectrometry (LC-MS/MS) analysis was used to quantify concentrations of bioactive lipids, including the D series resolvin precursor 17-HDHA in plasma samples. The method was adapted from that previously reported^69^ for the analysis of a wider range of oxylipins including 17-HDHA. Briefly, lipids were extracted from plasma samples via protein precipitation followed by solid-phase extraction. LC-MS/MS used negative ionisation mode and quantification was performed using the analyte to internal standard peak area ratio against a fully extracted calibration line. Endogenous (n=6) and spiked QC standards (n=6) were extracted and analysed across the sample run and met QC acceptance criteria of <15%CV.

#### Isolation of monocytes by fluorescence-activated cell sorting

Peripheral blood mononuclear cells (PBMCs) were isolated from whole blood with SepMate isolation tubes and Lymphoprep density gradient. PBMCs were then incubated with the following panel of antibodies; APC anti-human CD14 (Biolegend: 301807), Brilliant Violet 421 anti-human CD16 (Biolegend: 302037), PE anti-human CD66 (Biolegend: 342303), and PE/Cy7 anti-human HLA (Biolegend: 327017). Dead cells were excluded by staining with propidium iodide (Biolegend:421301). Flow cytometry and cell sorting was performed using a Beckman Coulter Astrios EQ cell sorter. Data were analysed with Kaluza Analysis software.

#### Bulk RNA sequencing and bioinformatic analysis

Total RNA was extracted from cell suspensions of classical monocytes (CD14++/CD16-/CD66b-/HLA-DR+) and intermediate monocytes (CD14++/CD16+/CD66b-/HLA-DR+) using TRIzol reagent. Next generation RNA sequencing was performed by Genewiz. Briefly, the SMART-Seq v4 Ultra Low Input RNA Kit (Clontech) was used to perform cDNA synthesis and amplification. Illumina-compatible sequencing libraries were constructed and then sequenced on the Illumina HiSeq 2500 with a 2 x 150 paired-end configuration.

Raw counts were processed by Genewiz prior to differential gene expression analysis and gene ontology analysis. The quality of raw counts was assessed using FASTQC and reads were subsequently trimmed to remove adapter sequence and low quality nucleotide calls using Trimmomatic v.0.36. STAR aligner v.2.5.2b was used to map the trimmed reads to the ENSEMBL Homo sapiens GRCh38 genome. Unique gene hit counts were obtained through the featureCounts tool of the Subread package v.1.5.2 with only unique reads falling within exon regions counted.

### Quantification and statistical analysis

Data were analysed using Graphpad PRISM (V9.0), Kaluza Analysis Software (V.2.1), R (V 4.2.0) (Bioconductor) and QIAGEN Ingenuity Pathway Analysis (IPA). Participant characteristics were analysed by either unpaired t-test or Mann Whitney U test. Differences in the proportion of monocyte populations were analysed by one-way ANOVA with Dunn’s multiple corrections. Gene ontology analysis was performed using GeneSCF v1.1-p2 and the goa_human GO list to cluster gene sets according to biological processes. *A priori* analysis was performed on genes identified as involved in the synthesis, metabolism, and signalling of 17-HDHA. Correlations between rlog transformed hit counts for *a priori* selected genes and pain or plasma concentrations of 17-HDHA were analysed by Spearman Rho, with correction for multiple comparison using false discovery rate.

Participants were stratified into groups based the median plasma levels of 17-HDHA (cut-off 0.5 nM) and self-reported pain scores (cut-off NRS of 6), consistent with previous studies.^65^ This resulted in four groups that were used for subsequent analyses: Low 17-HDHA-Low Pain (LH-LP) n=9; High 17-HDHA-Low Pain (HH-LP) n=8; Low 17-HDHA-High Pain (LH-HP) n=8; and High 17-HDHA-High Pain (HH-HP) n=5.

Differential gene expression analysis with DESeq2 compared subgroup data from the classical and intermediate monocyte populations separately. As this is a relatively small exploratory clinical study, this influenced the power of our study. As a result we used p values and log2 fold change cut off for the selection of differentially expressed genes to be inputted into the IPA analysis. This approach ensured we had the optimal number of differentially expressed genes for the main comparison of interest (HH-LP versus LH-HP) which form the focus of our study. The Wald test was used to generate p-values and log2 fold-changes. Differentially expressed genes (DEGs) were categorized as genes with adjusted p-value < 0.05 and absolute log2 fold change > 1.

QIAGEN Ingenuity Pathway Analysis^70^ was used to identify patterns of functional pathways in the transcriptional profile of the classical and intermediate monocytes for the four subgroups of participants (as defined above). All genes with measurable counts in at least one participant group, according to the normalisation carried out by Genewiz, were uploaded to IPA with data for log2 fold change, p-value and adjusted p-value. Cut-offs for inclusion in pathway analysis with IPA were imposed on log2 fold change +/-1 and p-value<0.05. P-value was considered rather than adjusted p-value to facilitate an exploratory analysis of the comparison of interest (HH-LP versus LH-HP). 805 genes fulfilled the criteria for pathway analysis for classical monocyte population and 388 genes for intermediate monocyte population.

## Supplemental information

Document S1. Figures S1–S3 and Tables S1 and S2

Data S1. Percentage of cells which are classical and intermediate monocytes from the total number of live cell, related to Figures 1 and S2 Percentage of cells which are classical and intermediate are shown for each of the four sub-group used for analysis.
