# Supplementary material for: Monocyte eukaryotic initiation factor 2 signaling differentiates 17-hydroxy-docosahexaenoic acid levels and pain
Source: iScience. 2025 Jan 21;28(2):111862. doi: 10.1016/j.isci.2025.111862 (PMC11848799; doi:10.1016/j.isci.2025.111862)
Supplement: Document S1. Figures S1–S3 and Tables S1 and S2 [file mmc1.pdf]

iScience, Volume *28*

Supplemental information

Monocyte eukaryotic initiation factor 2 signaling differentiates 17-hydroxy-docosahexaenoic acid levels and pain

Peter R.W. Gowler, Asta Arendt-Tranholm, James Turnbull, Rakesh R. Jha, David Onion, Tony Kelly, Afroditi Kouraki, Paul Millns, Sameer Gohir, Susan Franks, David A. Barrett, Ana M. Valdes, and Victoria Chapman

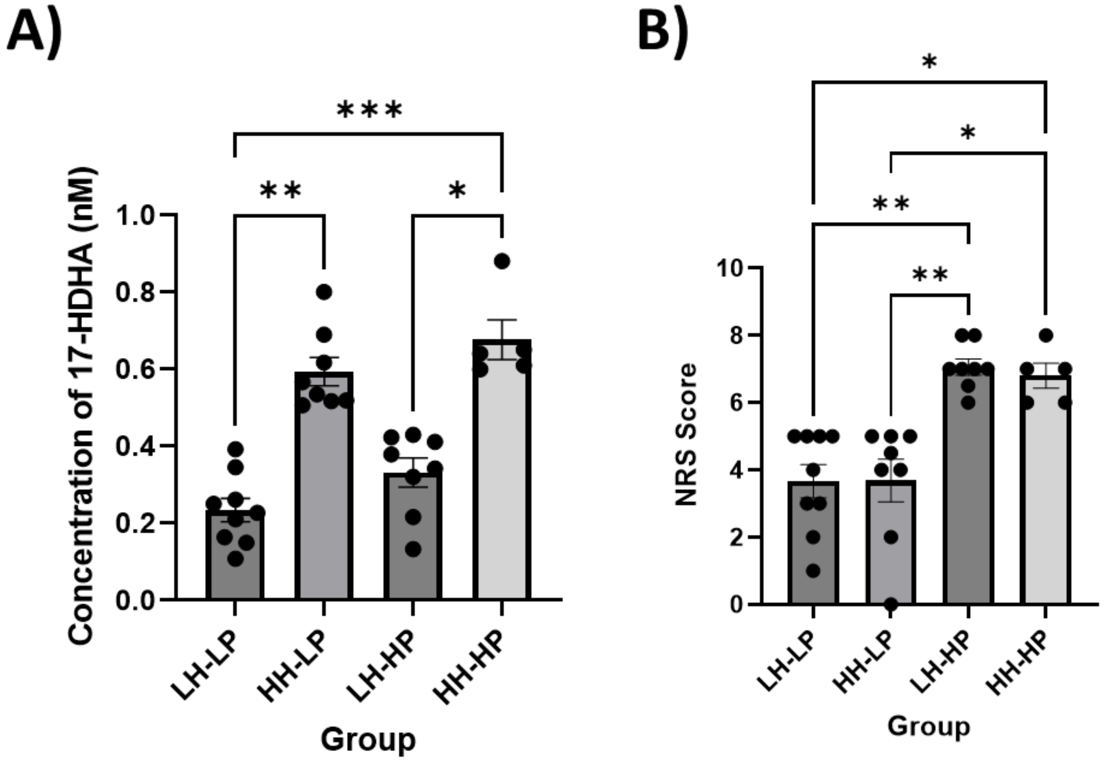

Supp.Figure 1: Analysis of key participant characteristics between groups. Comparison of (A) 17-HDHA levels and (B) NRS scores between participant groups stratified using the median concentration of 17-HDHA (0.5 nM) and median NRS Score 6 as cut-offs. Differences between groups were assessed using Kruskal-Wallis Test with multiple comparisons (corrected by Dunn's). Data are represented as mean +/- SEM with individual values also shown. *p=<0.05; **p=<0.01;***p=<0.001. LH-LP (Low 17-HDHA-Low Pain); HH-LP (High 17-HDHA-Low Pain); LH-HP (Low-17-HDHA-High Pain); HH-HP (High 17-HDHA-High Pain). Related to Figure 1.

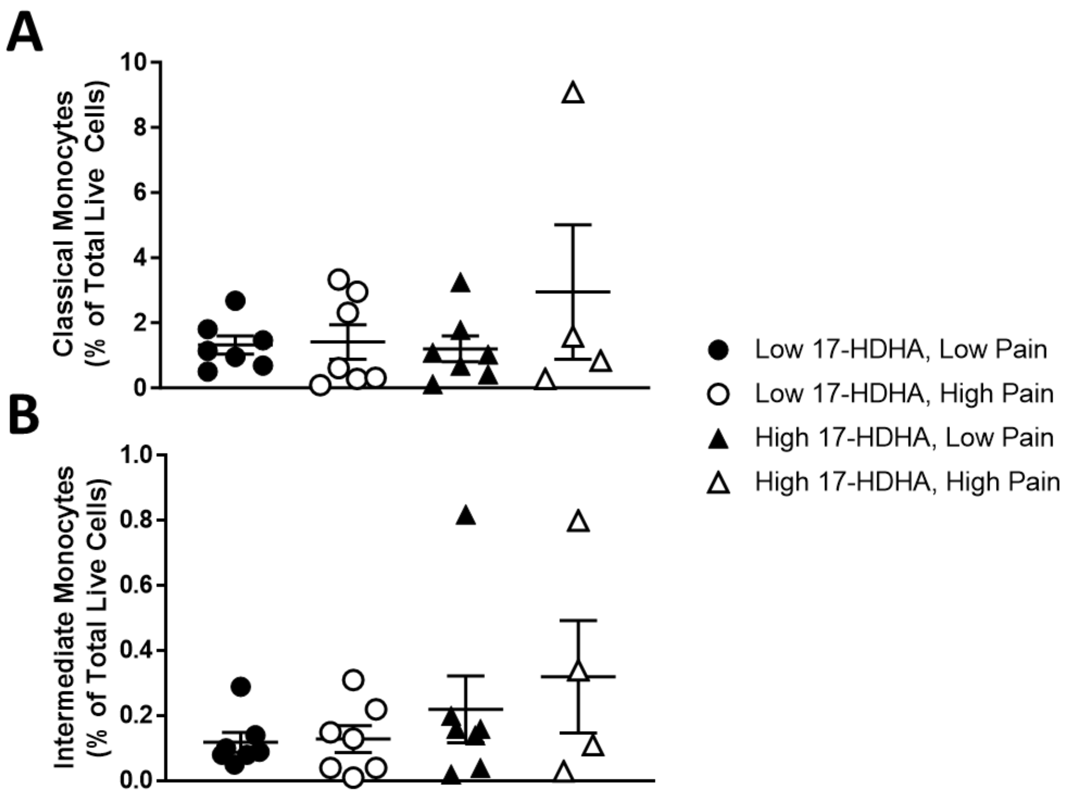

Supp.Figure 2: Percentage of total live cells in each group. The percentage of cells which are A) classical monocytes and B) intermediate monocytes from the total number of live cells, shown for each of the four sub-group used for analysis. Related to Figure 2. Data are represented as mean +/- SEM with individual values also shown.

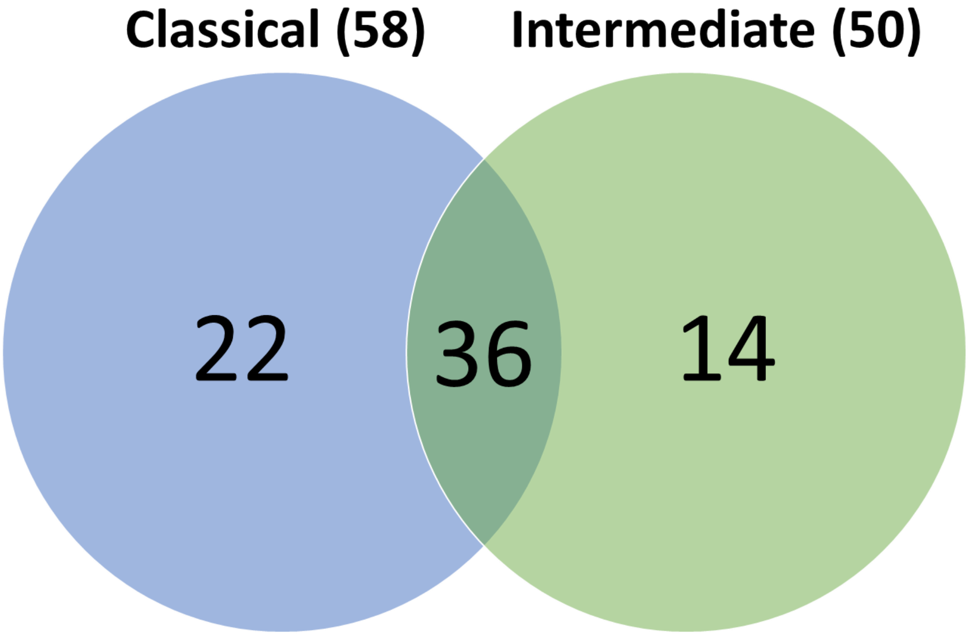

Supp Figure 3: The number of overlapping versus unique pathways identified by IPA analysis between the high 17-HDHA and low pain group and the low 17-HDHA and high pain group for the two populations of monocytes. Related to Table 6.

Group	Comparison	Total No. Pathway Identified by IPA
Classical Monocytes	HH, LP vs LH, HP	58
	HH, LP vs HH, HP	62
	HH, LP vs LH, LP	30
	HH, HP vs LH, HP	58
	HH, HP vs LH, LP	46
	LH, LP vs LH, HP	115
Intermediate Monocytes	HH, LP vs LH, HP	50
	HH, LP vs HH, HP	56
	HH, LP Vs LH, LP	63
	HH, HP vs LH, HP	41
	HH, HP vs LH, LP	32
	LH, LP vs LH, HP	37

Supp Table 1: The number of significant pathways identified by IPA for all comparisons for classical and intermediate monocytes. Related to Table 6.

Overlapping pathways	Classical	Intermediate
Communication between Innate and Adaptive Immune Cells	EIF2 Signaling	Kinetochore Metaphase Signaling Pathway
Hematopoiesis from Pluripotent Stem Cells	Regulation of eIF4 and p70S6K Signaling	Primary Immunodeficiency Signaling
Systemic Lupus Erythematosus Signaling	mTOR Signaling	Airway Inflammation in Asthma
CTLA4 Signaling in Cytotoxic T Lymphocytes	Coronavirus Pathogenesis Pathway	Phagosome Formation
Dendritic Cell Maturation	Basal Cell Carcinoma Signaling	CDC42 Signaling
PKCÎ¸ Signaling in T Lymphocytes	Nitric Oxide Signaling in the Cardiovascular System	Erythropoietin Signaling Pathway
Autoimmune Thyroid Disease Signaling	Human Embryonic Stem Cell Pluripotency	Sulfate Activation for Sulfonation
IL-15 Signaling	Oxidative Phosphorylation	Ascorbate Recycling (Cytosolic)
CCR5 Signaling in Macrophages	Iron homeostasis signaling pathway	Biotin-carboxyl Carrier Protein Assembly
SAPK/JNK Signaling	Regulation Of The Epithelial Mesenchymal Transition In Development Pathway	Estrogen-mediated S-phase Entry
TEC Kinase Signaling	NAD Signaling Pathway	Mitotic Roles of Polo-Like Kinase
ICOS-ICOSL Signaling in T Helper Cells	Axonal Guidance Signaling	Uracil Degradation II (Reductive)
Role of NFAT in Regulation of the Immune Response	Role of Osteoblasts, Osteoclasts and Chondrocytes in Rheumatoid Arthritis	Phenylethylamine Degradation I
B Cell Receptor Signaling	Estrogen Receptor Signaling	Thymine Degradation
Allograft Rejection Signaling	Clathrin-mediated Endocytosis Signaling	
Lipid Antigen Presentation by CD1	Macropinocytosis Signaling	
Cytotoxic T Lymphocyte-mediated Apoptosis of Target Cells	Sonic Hedgehog Signaling	
Antiproliferative Role of TOB in T Cell Signaling	Mitochondrial Dysfunction	
CD28 Signaling in T Helper Cells	Amyotrophic Lateral Sclerosis Signaling	
OX40 Signaling Pathway	Role of NANOG in Mammalian Embryonic Stem Cell Pluripotency	
Graft-versus-Host Disease Signaling	Hepatic Fibrosis Signaling Pathway	
Systemic Lupus Erythematosus In B Cell Signaling Pathway	Osteoarthritis Pathway	
NF-ÎºB Signaling		
Altered T Cell and B Cell Signaling in Rheumatoid Arthritis		
Calcium-induced T Lymphocyte Apoptosis		
Regulation of IL-2 Expression in Activated and Anergic T Lymphocytes		
T Helper Cell Differentiation		
Phospholipase C Signaling		
Cell Cycle Control of Chromosomal Replication		
Role of Macrophages, Fibroblasts and Endothelial Cells in Rheumatoid Arthritis		
Th17 Activation Pathway		
T Cell Exhaustion Signaling Pathway		
T Cell Receptor Signaling		
Type I Diabetes Mellitus Signaling		
NUR77 Signaling in T Lymphocytes		
Systemic Lupus Erythematosus In T Cell Signaling Pathway		

Supp Table 2: Description of the overlapping versus unique pathways identified by IPA analysis between the high 17-HDHA and low pain group and the low 17-HDHA and high pain group for the two populations of monocytes. Related to Table 6.
